# Synergistic Effect of Quercetin and *α*-Lipoic Acid on Aluminium Chloride Induced Neurotoxicity in Rats

**DOI:** 10.1155/2018/2817036

**Published:** 2018-05-16

**Authors:** Sooad Saud Al-Otaibi, Maha Mohamad Arafah, Bechan Sharma, Abdullah Salih Alhomida, Nikhat Jamal Siddiqi

**Affiliations:** ^1^Department of Biochemistry, College of Science, King Saud University, Riyadh, Saudi Arabia; ^2^Department of Pathology, College of Medicine, King Saud University, Riyadh, Saudi Arabia; ^3^Department of Biochemistry, University of Allahabad, Allahabad 211002, India; ^4^Department of Biochemistry, College of Science, P.O. Box 22452, King Saud University, Riyadh 11495, Saudi Arabia

## Abstract

**Objectives:**

The present study was carried out to study the protective effects of quercetin and *α*-lipoic acid alone and in combination against aluminum chloride induced neurotoxicity in rats.

**Materials and Methods:**

The study consisted of eight groups, namely, Group 1: control rats, Group 2: rats receiving aluminium chloride 7 mg/kg body weight intraperitoneal route (i.p) for two weeks, Group 3: rats receiving quercetin 50 mg/kg body weight i.p. for two weeks, Group 4: rats receiving quercetin 50 mg/kg body weight followed by aluminium chloride 7 mg/kg body weight i.p. for two weeks, Group 5: rats receiving *α*-lipoic acid 20 mg/kg body weight i.p. for two weeks, Group 6: rats receiving lipoic acid 20 mg/kg body weight followed by aluminium chloride 7 mg/kg body weight i.p. for two weeks, Group 7: rats receiving *α*-lipoic acid 20 mg/kg body weight and quercetin 50 mg/kg body weight i.p. for two weeks, and Group 8: rats receiving *α*-lipoic acid 20 mg/kg body weight and quercetin 50 mg/kg body weight followed by aluminium chloride 7 mg/kg body weight i.p. for two weeks. The animals were killed after 24 hours of the last dose by cervical dislocation.

**Results:**

Aluminium chloride treatment of rats resulted in significant increases in lipid peroxidation, protein carbonyl levels, and acetylcholine esterase activity in the brain. This was accompanied with significant decreases in reduced glutathione, activities of the glutathione reductase, and superoxide dismutase. Pretreatment of AlCl_3_ exposed rats to either quercetin or *α*-lipoic acid also restored altered lipid peroxidation and superoxide dismutase to near normal levels. Quercetin or *α*-lipoic acid pretreatment of AlCl_3_ exposed rats improved the protein carbonyl and reduced glutathione, glutathione reductase, and acetylcholine esterase activities in rat brains towards normal levels. Combined pretreatment of AlCl3 exposed rats with quercetin and *α*-lipoic acid resulted in a tendency towards normalization of most of the parameters.

**Conclusions:**

Quercetin and *α*-lipoic acid complemented each other in protecting the rat brain against oxidative stress induced by aluminium chloride.

## 1. Background

Aluminium is ubiquitously distributed as environmental and is an industrial toxicant. It is also present in many food products. It has been implicated in skeletal, haematological, and neurological diseases [1]. Aluminium mimics metals such as magnesium, calcium, and iron in their biological functions and thus causes biochemical alterations [2, 3]. Aluminium induces neurodegeneration, through an increase in Fe accumulation and reactive oxygen species production [3]. There are numerous studies which show that aluminium promotes iron-dependent lipid peroxidation in rat model [4, 5]. The neutralization of reactive oxygen species is an important strategy to prevent the beginning or progression of pathological processes related to metal intoxication. Quercetin is a polyphenolic flavonoid distributed in the plant kingdom and has been reported to possess antioxidant activity [6]. *α*-Lipoic acid (ALA) is another antioxidant that can cross the blood-brain barrier [7, 8]. ALA is an endogenous thiol antioxidant, which has effective potential to regenerate reduced glutathione, quench reactive oxygen species, and chelate metals such as iron, copper, cadmium, and mercury.

The present study was undertaken to investigate the protective effects of two antioxidants, namely, quercetin and ALA in aluminium chloride (AlCl_3_) induced neurotoxicity in rats.

## 2. Materials and Methods

### 2.1. Chemicals

All the chemicals were purchased from Sigma Chemical Co., St. Louis, MO, USA.

### 2.2. Animals

Healthy adult male Wistar rats weighing 200–250 g (four to six weeks old) were obtained from the animal house of King Saud University, Riyadh, Saudi Arabia. The animals were labeled by identifying ear notches, housed in clean cages, and placed in the animal care room. Rats were allowed free access to food (Purina rodent chow) and tap water. Ethical animal care guidelines were followed. This study was approved by the animal ethics committee of College of Science, King Saud University, Riyadh, Saudi Arabia (approval number: 4/67/389683).

### 2.3. Treatment

The following experimental groups of rats were studied. All the groups consisted of 16 rats of almost the same age and weight. The rats were injected with *α*-lipoic acid (ALA)/aluminium chloride (AlCl_3_)/quercetin through intraperitoneal route (ip). Group 1 included control rats, Group 2 included rats receiving AlCl_3_ at a dose of 7 mg/kg body weight for two weeks, Group 3 included rats receiving quercetin at a dose of 50 mg/kg body weight for two weeks, Group 4 included rats receiving quercetin at a dose of 50 mg/kg body weight followed by AlCl_3_ 7 mg/kg body weight for two weeks, Group 5 included rats receiving ALA 20 mg/kg body weight for two weeks, Group 6 included rats receiving ALA 20 mg/kg body weight followed by AlCl_3_ 7 mg/kg body weight for two weeks, Group 7 included rats receiving ALA 20 mg/kg body weight and quercetin 50 mg/kg body weight for two weeks, and Group 8 included rats receiving ALA 20 mg/kg body weight and quercetin 50 mg/kg body weight followed by AlCl_3_ 7 mg/kg body weight for two weeks. The animals were killed after 24 hours of the last dose by cervical dislocation.

### 2.4. Preparation of Sample

The brains were dissected out from the rats, cleared of the adhering tissues, weighed, and homogenized in normal saline (10% W/V). The homogenates were centrifuged at 3000*g* for 10 minutes. The resulting supernatants were used for biochemical assays.

### 2.5. Biochemical Assays

Lipid peroxidation was determined by the method of Utley et al., 1967 [9]. Protein carbonyl level in the samples was determined by the method Levine et al., 1990 [10]. Reduced glutathione was estimated by the method of Beutler et al., 1963 [11]. Glutathione reductase activity was assayed by the method of Goldberg and Spooner 1987 [12]. Superoxide dismutase was estimated by the method of Kakkar et al., 1984 [13]. Catalase was assayed by the method Aebi 1984 [14]. The activity of acetyl choline esterase was determined according to the method of Ellman et al., 1961 [15]. The protein content in the sample was measured by the method of Markwell et al., 1978 [16], using bovine serum albumin as the standard.

### 2.6. Preparation of the Sample for Light Microscopy

The brain was removed and fixed in 10% formalin and stored till further processed for microscopy. Tissues were processed to obtain 5 *μ*m thick paraffin wax, stained with haematoxylin and eosin according to the method of Bancroft and Stevens, 1996 [17], and used for histopathological examination.

### 2.7. Statistical Analysis

Quantitative data were statistically represented in terms minimum, maximum, mean, and standard division (SD). Comparisons before and after the treatment were done using paired samples *T*-test for comparing two parametric readings for the same group. One-way ANOVA test was used when more than two groups were compared and comparison between the groups was done using Dunnett's multiple comparison test. Correlation between various variables was done using Person correlation coefficient (*R*) and graphic representations were done using linear regression. A probability value (*p* value) less than or equal to 0.05 was considered significant. All statistical calculations were done using computer program SPSS (Statistical Package for Social Science) statistical program version (16.0). Graphs were prepared using SPSS statistical program version (16.0) (Correlation Graphs) and InStat® package for personal computers (Graph Pad TM Software, Inc., San Diego, USA, for means with error bars graphs).

## 3. Results

Table 1 shows the effect of AlCl_3_, quercetin, and ALA treatment alone and in combination on oxidative stress indices in rat brains. AlCl_3_ treatment caused a significant increase of 242% in lipid peroxidation of brain when compared with that of control rats (*p* < 0.001). Treatment of rats with quercetin followed by AlCl_3_ however restored the elevated lipid peroxidation to near normal levels (*p* > 0.05). Similarly pretreatment of AlCl_3_ exposed rats with ALA restored the increased lipid peroxidation to near normal levels in the brain of rats. Quercetin and ALA by themselves caused no significant changes in lipid peroxidation in rat brains (*p* > 0.05). Pretreatment of AlCl_3_ exposed rat with both quercetin and ALA also restored the elevated lipid peroxidation in brain to normal values.

AlCl_3_ treatment of rats resulted in a significant increase of 119% in the protein carbonyl levels of brains in rats when compared to control rats (*p* < 0.001). Quercetin and ALA caused significant decreases of 14% and 12% (*p* < 0.05), respectively, in protein carbonyl when compared to protein carbonyl level in the brain of control rats. Treatment of rats with either quercetin or ALA followed by AlCl_3_ resulted in significant decrease (*p* < 0.001) in brain protein carbonyl level when compared to that in AlCl_3_ alone in treated rats. Combined treatment of quercetin and ALA to AlCl_3_ treated rats resulted in restoration of protein carbonyl to almost normal levels.

Table 2 shows the effect of AlCl_3_, quercetin, and ALA treatment alone and together on various antioxidant defence indices in the brain of rats. AlCl_3_ treatment of rats caused a significant decrease of reduced glutathione by 50% when compared to control group (*p* < 0.001). Quercetin treatment also caused a decrease in reduced glutathione content of brain by 9% (*p* < 0.05) when compared to control rats. ALA on the other hand caused no significant alterations on the reduced glutathione content of rat brain. Although quercetin followed by AlCl_3_ treatment significantly increased reduced glutathione level when compared to AlCl_3_ alone group, it could not restore the brain GSH content to normal level. Though ALA by itself caused no significant change in reduced glutathione concentration, pretreatment of AlCl_3_ exposed rats with ALA resulted in significant increase (*p* < 0.001) of reduced glutathione when compared to AlCl_3_ treated rats. Quercetin and ALA when administered before AlCl_3_ restored the brain reduced glutathione to almost normal levels.

Administration of AlCl_3_ to rats caused a highly significant (*p* < 0.001) decrease in glutathione reductase activity by 44% when compared to that in the brain of control rats. Quercetin and ALA treatment to control rats caused no significant alterations in the glutathione reductase activity of rat brain. Quercetin and ALA pretreatment individually and together with AlCl_3_ injected rats caused significant increase (*p* < 0.05) in brain glutathione reductase activity when compared to AlCl_3_ treated group. However, they could not restore the glutathione reductase activity in rat brain to normal levels.

AlCl_3_ treatment of rats resulted in a significant decrease in superoxide dismutase activity in the brain of rats by 32% (*p* < 0.001) when compared to brain superoxide dismutase activity of control rats. Quercetin treatment of control rats resulted in significant increase of 11% (*p* < 0.05) in brain superoxide dismutase activity when compared to control rat brains. Quercetin pretreatment of AlCl_3_ injected rats resulted in restoration of brain superoxide dismutase activity to normal levels. Although ALA caused no significant (*p* > 0.05) alteration in the brain superoxide dismutase of control rats, it restored the AlCl_3_ altered superoxide dismutase activity to normal value. Combined treatment of quercetin, *α*-lipoic acid, and AlCl_3_ resulted in a significant increase of brain superoxide dismutase activity by 19% (*p* < 0.001) when compared to control rats.

Administration of AlCl_3_ to rats caused a highly significant (*p* < 0.001) increase of 106% in the brain catalase activity when compared to control rats. Quercetin and ALA treatment of control rats resulted in significant decrease in brain catalase activity by of 15% (*p* < 0.05) and 26% (*p* < 0.001), respectively, when compared to that in control rats. Pretreatment of AlCl_3_ exposed rats with quercetin restored brain catalase activity to normal level. ALA pretreatment of AlCl_3_ exposed rats significantly (*p* < 0.001) decreased the brain catalase activity below normal levels. Combined pretreatment of AlCl_3_ treated rats with quercetin plus *α*-lipoic acid could not restore the catalase activity in brain to normal level.

**Figure 1:**
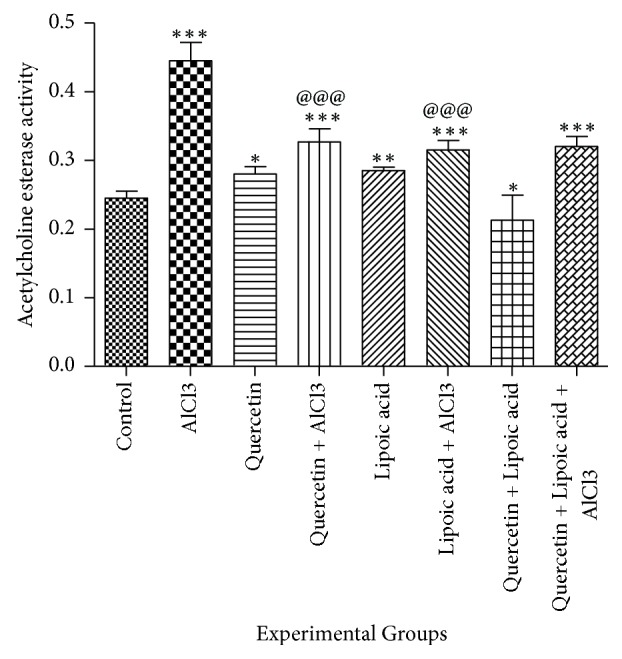
The effect of aluminium chloride, quercetin, and lipoic acid treatment on acetylcholine esterase activity in the brain of rats. Enzyme activity is expressed as nmoles of substrate hydrolyzed min^−1 ^mg protein^−1^ in the brain of rats. ^*∗∗∗*^Significant as compared to control group of rats (*p* < 0.001). ^*∗∗*^Significant as compared to control group of rats (*p* < 0.01). ^*∗*^Significant as compared to control group of rats (*p* < 0.05). ^@@@^Significant as compared to AlCl_3_ treated group of rats (*p* < 0.001). Comparison between the groups was done using Dunnett's multiple comparison test.

Figure 1 shows the effect of aluminium chloride, quercetin, and lipoic acid treatment alone and in combination on acetylcholine esterase activity in the brain of rats. AlCl_3_ treatment caused a significant increase in brain acetylcholine esterase activity by 82% (*p* < 0.001) when compared to normal rat brains. Quercetin and lipoic acid treatment also resulted in an increase of 14% (*p* < 0.05) and 16% (*p* < 0.01), respectively, in brain acetylcholine esterase activity when compared to control rats. Quercetin treatment followed by AlCl_3_ caused a significant decrease (*p* < 0.001) in brain acetylcholine esterase activity when compared to AlCl_3_ treated group. Similarly *α*-lipoic acid treatment followed by AlCl_3_ also caused a decrease in brain acetylcholine esterase when compared to AlCl_3_ treated group (*p* < 0.001). Combined treatment with quercetin, *α*-lipoic acid and AlCl_3_ resulted in a significant decrease (*p* < 0.001) in brain acetylcholine esterase when compared to AlCl3 treated group.

**Figure 2:**
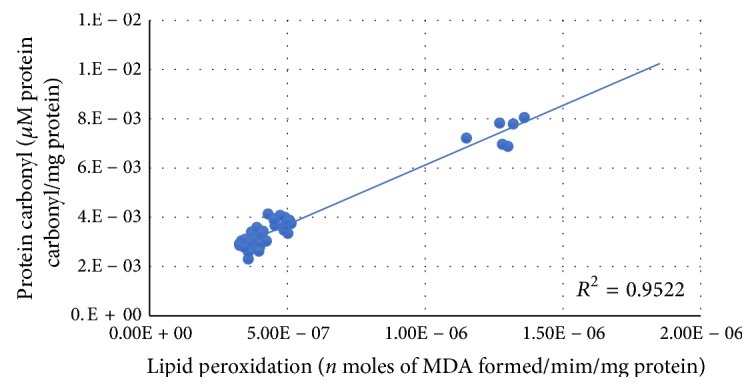
Correlation between lipid peroxidation and protein carbonyl with best fit line curve (positive correlation).

Figure 2 shows that the correlation was observed between lipid peroxidation and protein carbonyl. A positive correlation was observed between lipid peroxidation and protein carbonyl.

**Figure 3:**
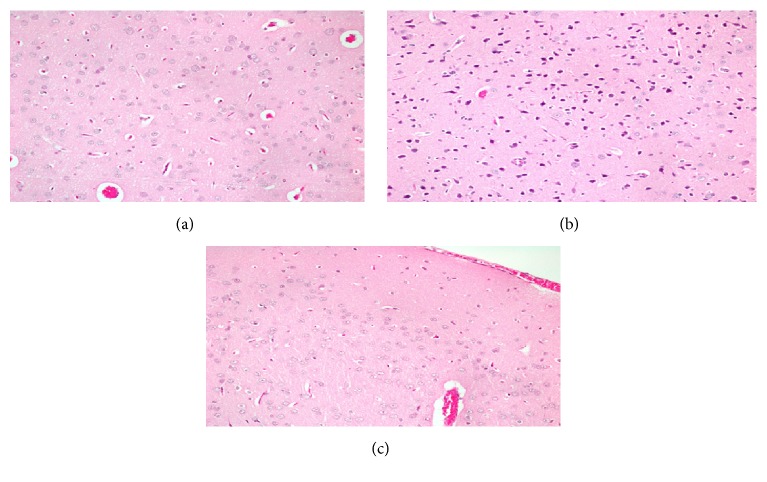
(a) Histopathological image of section of the brain of a control rat. The image shows normal cells in the brain. (b) Histopathological image of section of the brain of rat treated with AlCl_3_. It shows numerous small dark cells with no nucleus which are apoptotic cells. (c) Histopathological image of section of the brain of AlCl3 exposed rats pretreated with quercetin and *α*-lipoic acid treated rat. It shows normal cells with fewer apoptotic cells.

Figure 3(a) shows histopathological image of section of the brain of a control rat. The image shows normal cells in the brain.

Figure 3(b) shows histopathological image of section of the brain of rat treated with AlCl_3_. It shows numerous small dark cells with no nucleus which are apoptotic cells.

Figure 3(c) shows histopathological image of section of the brain of AlCl_3_ exposed rats pretreated with quercetin and *α*-lipoic acid treated rat. It shows normal cells with fewer apoptotic cells.

## 4. Discussion

Aluminum is a widely used household metal, which is associated with bone, blood, and brain diseases [18]. Aluminum has been shown to accumulate in all regions of the brain following chronic exposure [19]. Evidence suggests that the deposition of aluminum is associated with the pathophysiology of various neurodegenerative diseases like Alzheimer's disease, Parkinson's disease, dementia, and so on [20]. Aluminum has been shown to be responsible for critical neuropathologic lesions in Alzheimer's disease and other related disorders due to its ability to cross-link hyperphosphorylated proteins [21]. Aluminum has also been detected in amyloid fibers in the cores of senile plaques in brains of Alzheimer's disease patients [22]. The presence of aluminum in biological systems leads to prooxidant activity by promoting superoxide anion generation through Fenton reaction [23]. Brain tissues are highly susceptible to oxidative damage due to their high rate of oxygen consumption rate (20%), the presence of abundant polyunsaturated fatty acids in cell membranes, high iron content, and low antioxidative enzyme activities [24]. ALA is both fat and water soluble and can cross the blood brain barrier. ALA alleviates the toxic effect of reactive oxygen species in the brain by its antioxidant properties [25]. Quercetin, a flavonoid found in fruits and vegetables, has unique biological properties, namely, anticarcinogenic, anti-inflammatory, ability to inhibit lipid peroxidation, and platelet aggregation. ALA, usually found in small amounts in meats and vegetables, has potent antioxidant capacity [26, 27]. Previous studies have shown that ALA and its reduced form dihydrolipoic acid have an amphiphilic property that allows them to easily cross the blood brain barrier and cell membranes and helps to activate other antioxidants such as vitamin C, vitamin E, coenzyme Q10, and ubiquinone [1, 28].

In this study, lipid peroxidation which is one of the key indicators of oxidative stress was increased in the brain of AlCl_3_ treated rats. In brain tissue aluminum salts have been reported to induce lipid peroxidation by increasing the whole brain thiobarbituric acid reactive substances [29]. Lipid peroxidation products interfere with the brain* *homeostasis* *between inhibitory and excitatory neurons [30] and cause mitochondrial dysfunction in the brain [31, 32]. Quercetin possess strong antioxidant capacities due to its ability to scavenge free radicals and bind transition metal ions, thereby inhibiting lipid peroxidation [6]. Ohtawa et al. 1983 [33] proposed that binding of aluminium with transferrin reduces the binding of iron with this protein. This free intracellular iron may then stimulate the peroxidation of membrane lipids, resulting in membrane damage. Quercetin and ALA acting as a free radical scavenger may contribute to the reduction in the peroxidation of lipids. ALA has been shown to protect hippocampus against oxidative stress, by inhibiting the oxidation of lipids and protein [34]. In this study quercetin and lipoic acid individually and together restored AlCl_3_ altered lipid peroxidation to normal levels which may be attributed to their antioxidant properties.

Another index to measure oxidative stress is the oxidant status of proteins. Reactive oxygen species cause cellular damage by oxidizing amino acid residues on proteins and forming protein carbonyls [35]. In this study AlCl_3_ caused a significant increase in protein carbonyl levels in rat brain. Similar results have been reported by Kowalczyk et al., 2004 [36].* * Being antioxidants, both quercetin and lipoic acid treatment individually caused significant decreases in protein carbonyl content of AlCl_3_ treated rat brain but could not restore it to normal levels. However, combined treatment with quercetin and lipoic acid restored the protein carbonyl to normal levels. Since the protein carbonyl content correlates with the severity of pathogenesis [35], the results of this study indicate that a combination of quercetin and lipoic acid may provide a better antidote to aluminum toxicity.

Glutathione, glutathione reductase, superoxide dismutase, and catalase form the first line of cellular defence against free radical assault. In this study AlCl_3_ treatment resulted in a significant decrease in reduced glutathione content in the brain of rats. This was accompanied with a concomitant decrease in glutathione reductase activity. Bharathi et al., 2006 [37], have attributed these changes to reduced axonal mitochondria turnover, disruption of Golgi, or reduction of synaptic vesicles induced by AlCl_3_ treatment. Studies of Farr et al., 2012 [38], have demonstrated that alpha lipoic acid significantly improves reduced glutathione in the mice brain and at the same time decreases lipid peroxidation indicating a reversal of oxidative stress. In this study, quercetin and ALA pretreatment regenerated reduced glutathione in rat brain. But a combined pretreatment of quercetin and ALA proved to be a better than individual treatment. This may be attributed to ALA which is known to regenerate other antioxidants such as glutathione, vitamin E, vitamin C, and coenzyme Q10 [39].

In the present study, AlCl_3_ was found to significantly inhibit superoxide dismutase activity in rat brain. This inhibition may be the result of inhibition of superoxide dismutase by superoxide anions [40]. Quercetin and lipoic acid pretreatment of AlCl_3_ exposed rats restored the altered superoxide dismutase to normal levels. However, combined pretreatment of AlCl_3_ exposed rats with quercetin and lipoic acid seemed to exhibit a synergetic effect. Studies of Sharma et al., 2013 [41], have shown that quercetin restores the AlCl_3_ inhibited superoxide dismutase to normal levels. They attributed it to the antioxidant properties of quercetin. The synergistic effect of quercetin and lipoic acid pretreatment on superoxide dismutase may be attributed to upregulation of Mn superoxide dismutase gene [42]. This result probably can be explained by the effect of ALA on nerve growth factor. There is a possibility that ALA enhances nerve growth factor-induced regulation of superoxide dismutase gene. This could be a factor leading to an increase in superoxide dismutase activity [39, 43].

Catalase is an antioxidant enzyme which inhibits oxidative damage by catalyzing the decomposition of hydrogen peroxide to water and oxygen [44]. Though earlier studies [45, 46] have shown AlCl_3_ to inhibit catalase activity in rat brain, in the present study there was an increase in brain catalase activity in AlCl_3_ treated rats. This variation may be explained by differential effects of oxidative stress on catalase activities in various regions of brain [47]. Studies of Singla and Dhawan 2014 [48] have shown that AlCl_3_ causes an increase in catalase activity in rat brain. However, quercetin pretreatment of AlCl_3_ treated rats restored catalase activity to almost normal level. ALA pretreatment of AlCl3 exposed rats did not restore altered catalase activity to normal level. Quercetin has been shown to protect rat brain against endosulfan induced oxidative stress [49]. ALA, on the other hand, could not restore the altered catalase activity to normal value.

In the present study, AlCl_3_ was shown to increase acetylcholine esterase activity. AlCl_3_ has been shown to increase acetylcholine esterase activity in rat and mice brain [50, 51]. AlCl_3_ has been shown to cause a biphasic effect on the acetylcholinesterase activity, with an initial increase in the activity of this enzyme during 4–14 days of exposure followed by a marked decrease. This has been attributed to the slow accumulation of aluminium in the brain [52]. The increase in acetylcholine esterase activity after AlCl_3_ treatment observed in the present study may be the initial phase of response of the brain to AlCl_3_ assault. Quercetin and lipoic acid caused significant reduction in acetylcholine esterase activity in the brain of AlCl_3_ treated rats. In vitro quercetin has been shown to exhibit acetylcholine esterase inhibitory activity along with Fe^2+^ chelating activity [53].

The increased vulnerability of the AlCl_3_ treated rat brain to oxidative stress as shown by increased oxidative stress and decreased antioxidant enzymes was mirrored in histopathological findings. The brain of AlCl_3_ treated rat showed numerous dark cells with no nucleus which were most likely apoptotic cells. However, the brains of AlCl_3_ exposed rats which were pretreated with either quercetin of alpha lipoic acid or both showed fewer apoptotic cells.

## 5. Conclusion

The present study concludes that both quercetin and alpha lipoic acid complement each other in protecting the rat brain against oxidative stress induced by aluminium chloride.

## Figures and Tables

**Table 1:** Effect of aluminum chloride, quercetin, and *α*-lipoic acid (ALA) treatment on oxidative stress indices in rat brain.

Experimental groups	Lipid peroxidation^a^	Protein carbonyl^b^
Control	4.21 ± 0.61	34.0 ± 2.39
Aluminum chloride	14.4 ± 0.2^*∗∗∗*^	74.6 ± 4.98^*∗∗∗*^
Quercetin	3.7 ± 0.21^NS^	29.4 ± 1.25^*∗*^
Quercetin + aluminum chloride	4.72 ± 0.26^NS^	38.5 ± 2.37^*∗*@^
*α*-Lipoic acid	3.67 ± 0.249^NS^	29.8 ± 1.17^*∗*^
*α*-Lipoic acid + aluminum chloride	4.77 ± 0.272^NS^	38.2 ± 1.59^*∗*@^
Quercetin + *α*-lipoic acid	3.58 ± 0.23^NS^	29.69 ± 2.32^*∗∗∗*^
Quercetin + *α*-lipoic acid + aluminum chloride	4.14 ± 0.07^NS^	32.7 ± 1.81^NS^

^a^Values are expressed as ± nmoles × 10^−7^ of MDA formed/min/mg protein; ^b^values are expressed as ± *μ* M protein carbonyl/mg protein; ^*∗∗∗*^significant as compared to control group of rats (*p* < 0.001); ^*∗*^significant as compared to control group of rats (*p* < 0.05); ^NS^nonsignificant as compared to control (*p* > 0.05); ^@^significant as when compared to AlCl_3_ (*p* < 0.05); comparison between the groups was done using Dunnett's multiple comparison test.

**Table 2:** Effect of aluminum chloride, quercetin, and *α*-lipoic acid (ALA) treatment on various antioxidant defence indices in the brain of rats.

Experimental groups	Reduced glutathione^a^	Glutathione reductase^b^	Superoxide dismutase^c^	Catalase^d^
Control	0.268 ± 0.024	0.062 ± 0.003	1.33 ± 0.14	2.68 ± 0.30
Aluminum chloride	0.135 ± 0.011^*∗∗∗*^	0.035 ± 0.004^*∗∗∗*^	0.90 ± 0.07^*∗∗∗*^	5.52 ± 0.34^*∗∗∗*^
Quercetin	0.243 ± 0.015^*∗*^	0.065 ± 0.005^NS^	1.47 ± 0.11^*∗*^	2.28 ± 0.25^*∗*^
Quercetin + aluminum chloride	0.223 ± 0.017^*∗∗∗*@@@^	0.052 ± 0.001^*∗∗∗*@@@^	1.36 ± 0.05^NS^	2.68 ± 0.20^NS@@@^
*α*-Lipoic acid	0.258 ± 0.019^NS^	0.063 ± 0.004^NS^	1.41 ± 0.03^NS^	1.97 ± 0.09^*∗∗∗*^
*α*-Lipoic acid + aluminum chloride	0.223 ± 0.015^*∗∗∗*@@@^	0.056 ± 0.004^*∗*@@@^	1.28 ± 0.04^NS^	1.96 ± 0.16^*∗∗∗*@@@^
Quercetin + *α*-lipoic acid	0.290 ± 0.014^NS^	0.062 ± 0.004^NS^	1.74 ± 0.19^*∗∗∗*^	2.59 ± 0.26^NS^
Quercetin + *α*-lipoic acid + aluminum chloride	0.247 ± 0.014^NS^	0.052 ± 0.001^*∗∗∗*^	1.58 ± 0.05^*∗∗∗*^	2.22 ± 0.22^*∗∗*^

^*∗∗∗*^Significant as compared to control group of rats (*p* < 0.001). ^*∗∗*^Significant as compared to control group of rats (*p* < 0.01). ^*∗*^Significant as compared to control group of rats (*p* < 0.05). ^@@@^Significant as compared to AlCl_3_ treated group of rats (*p* < 0.001). ^NS^Nonsignificant as compared to control (*p* > 0.05). Comparison between the groups was done using Dunnett's multiple comparison test; ^a^values are expressed as ±mg of reduced glutathione mg protein^−1^; ^b^values are expressed as ±millimoles of glutathione reduced min^−1 ^mg protein^−1^; ^c^values are expressed as ±units mg protein^−1^. One unit of enzyme was defined as the amount of enzyme which inhibited formazone formation by 50%; ^d^values are expressed as ± *μ*mole of H_2_O_2_ dissipated min^−1 ^mg protein^−1^.

## Data Availability

Authors can confirm that all relevant data are included in the article and/or its supplementary information files.
